# Y Chromosomal Variation Tracks the Evolution of Mating Systems in Chimpanzee and Bonobo

**DOI:** 10.1371/journal.pone.0012482

**Published:** 2010-09-01

**Authors:** Felix Schaller, Antonio M. Fernandes, Christine Hodler, Claudia Münch, Juan J. Pasantes, Wolfram Rietschel, Werner Schempp

**Affiliations:** 1 Institute of Human Genetics, University of Freiburg, Freiburg, Germany; 2 Department of Biochemistry, Genetics and Immunology, University of Vigo, Vigo, Spain; 3 Wilhelma der zoologisch botanische Garten, Stuttgart, Germany; Max Planck Institute for Evolutionary Anthropology, Germany

## Abstract

The male-specific regions of the Y chromosome (MSY) of the human and the chimpanzee (*Pan troglodytes*) are fully sequenced. The most striking difference is the dramatic rearrangement of large parts of their respective MSYs. These non-recombining regions include ampliconic gene families that are known to be important for male reproduction,and are consequently under significant selective pressure. However, whether the published Y-chromosomal pattern of ampliconic fertility genes is invariable within *P. troglodytes* is an open but fundamental question pertinent to discussions of the evolutionary fate of the Y chromosome in different primate mating systems. To solve this question we applied fluorescence *in situ* hybridisation (FISH) of testis-specific expressed ampliconic fertility genes to metaphase Y chromosomes of 17 chimpanzees derived from 11 wild-born males and 16 bonobos representing seven wild-born males. We show that of eleven *P. troglodytes* Y-chromosomal lines, ten Y-chromosomal variants were detected based on the number and arrangement of the ampliconic fertility genes *DAZ* (deleted in azoospermia) and *CDY* (chromodomain protein Y)—a so-far never-described variation of a species' Y chromosome. In marked contrast, no variation was evident among seven Y-chromosomal lines of the bonobo, *P. paniscus*, the chimpanzee's closest living relative. Although, loss of variation of the Y chromosome in the bonobo by a founder effect or genetic drift cannot be excluded, these contrasting patterns might be explained in the context of the species' markedly different social and mating behaviour. In chimpanzees, multiple males copulate with a receptive female during a short period of visible anogenital swelling, and this may place significant selection on fertility genes. In bonobos, however, female mate choice may make sperm competition redundant (leading to monomorphism of fertility genes), since ovulation in this species is concealed by the prolonged anogenital swelling, and because female bonobos can occupy high-ranking positions in the group and are thus able to determine mate choice more freely.

## Introduction

Published cytogenetic comparisons clearly show size differences among the Y chromosomes of our nearest relatives, the chimpanzee (*Pan troglodytes*) and the bonobo (*Pan paniscus*) [1], [2]. The chimpanzee Y chromosome is the smallest of the complement and is almost metacentric in morphology, while the bonobo Y is submetacentric, and similar in size to the G-group chromosomes of this species. The difference is attributable to a large early replicating euchromatic segment present in the proximal long arm of the bonobo Y [1], [3] that is absent in the chimpanzee. Additionally, both the chimpanzee and the bonobo Y chromosomes exhibit a C-band positive heterochromatic segment at the tip of their short arms (Yp) – a characteristic feature of the distal long arm of the human Y chromosome (Yq). The pseudoautosomal region (PAR), together with the sex-determining region on the Y (*SRY*) located at the tip of human Yp, is located at the tip of Yq in chimpanzee and bonobo. Single-copy genes (X-degenerated genes), that map to human Yp and proximal Yq, and which survive as relicts from ancient autosomes from which the X and Y evolved [4], are shown to be conserved and arranged as single-copy genes along the distal half of the chimpanzee and bonobo Yq [3], [5]. The non-recombining ampliconic fertility genes *TSPY* and *RBMY* are shown to be highly amplified on the bonobo Y when compared to the chimpanzee Y, while these are significantly rearranged on the human Y [2], [3]. The male-specific regions of the Y chromosome (MSY) of the human and the chimpanzee (*P. troglodytes*) are fully sequenced now [6]–[9]. Comparison of the MSYs of the two species has shown dramatic rearrangements especially in the non-recombining parts that harbour ampliconic and repeated fertility genes [9]. Comparable data from the MSY of the bonobo (*P. paniscus*) the common chimpanzee's closest relative are still missing. However, such data from bonobo as an outgroup species are essential to draw conclusions on the evolutionary fate of the Y chromosome in different primate species. Interestingly, a recent study showed intra-species variation in the copy number of the Y-specific ampliconic fertility gene *DAZ* within chimpanzee, but not bonobo [10]. The restriction of *DAZ*-variation to chimpanzee prompted us to scrutinize the variability of the non-recombining part of the Y chromosome in a number of chimpanzees and bonobos more closely. Our results disclose a high intra-species variation in number and arrangement of ampliconic fertility genes among chimpanzee Y chromosomes while no variation was evident among bonobo Y chromosomes.

## Results and Discussion

**Figure 1:**
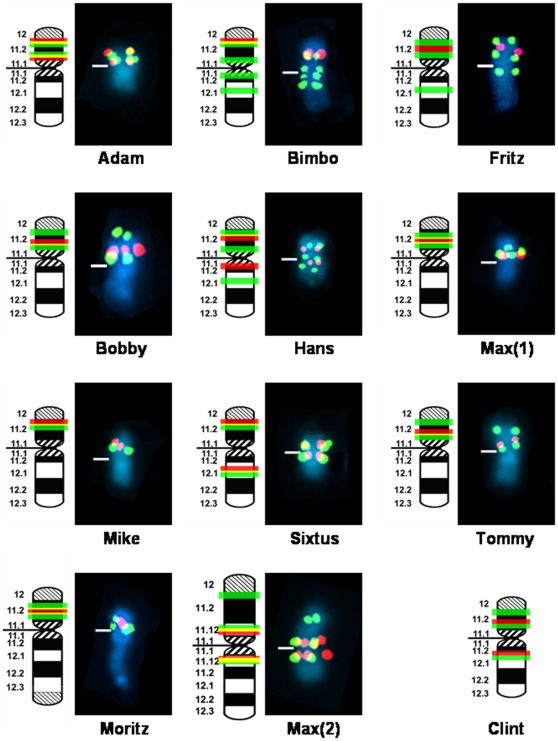
FISH mapping of ampliconic genes *DAZ* and *CDY* in eleven Y chromosome lineages of chimpanzee. FISH signal patterns for *DAZ* (red) and *CDY* (green) is shown for each Y chromosome lineage included in the present investigation. The band locations of each gene are shown on the ideogram to the left of each FISH image. Yellow signals results from overlapping of red and green signals. Centromeres are marked by horizontal lines. Band nomenclature follows the ISCN [49]. The diagrammatic *DAZ* (red) and *CDY* (green) signal pattern for “Clint” is deduced from Hughes et al. [9] identifying a further Y variant in chimpanzee (see text).

**Figure 2:**
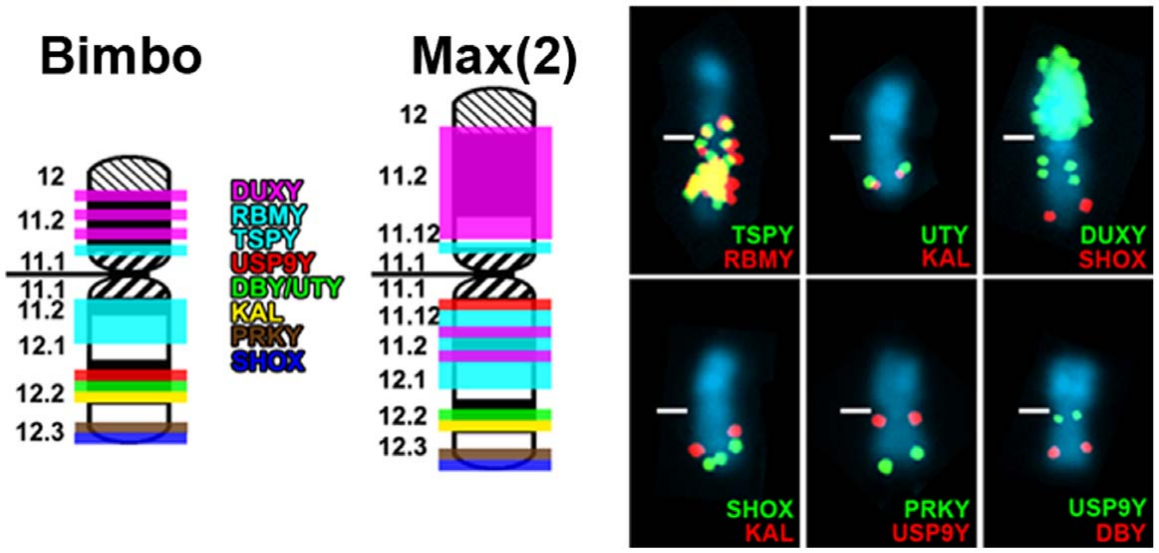
Comparative FISH mapping of ampliconic and X-degenerate genes on the Y chromosomes of “Max(2)” and “Bimbo”. On the right are shown the comparative FISH results for ampliconic Y chromosome genes *TSPY*, *RBMY* and *DUXY*, for the X-degenerate Y chromosome genes *UTY*, *KAL*, *PRKY*, *USP9Y* and *DBY* (now named *DDX3Y*), and for the pseudoautosomal gene *SHOX*. Centromeres are marked by white bars. On the left is shown a diagram with the FISH signal patterns for the Y chromosomes of “Max(2)” compared to “Bimbo”. Band nomenclature follows the ISCN [49].

Our focus was initially directed at the structural arrangement of the ampliconic fertility genes *DAZ* and *CDY*, both of which are expressed exclusively in the testis of human [6] and chimpanzee [9]. As a consequence, we mapped human-derived DNA probes specific for *DAZ* and *CDY* to metaphase Y chromosomes of 17 chimpanzees derived from 11 wild-born males, and 16 bonobos representing seven wild-born males by fluorescence *in situ* hybridization (FISH) (Figures S1 and S2; Tables S1 and S2). Our results revealed highly diverse signal copy numbers and Y-chromosomal locations for *DAZ* and *CDY* genes for the chimpanzees (Figure 1). We detected ten Y-chromosomal variants among the 11 male chimpanzee lineages represented in our investigation. Only “Bobby” and “Tommy”, both wild-born chimpanzees (Table S1), presented the same Y chromosome. Furthermore, two wild-born chimpanzees, “Max(1)” and “Moritz” (Table S1), exhibited an identical pattern for *DAZ* and *CDY* but, importantly, the “Moritz” Y chromosome differed by a pericentromeric inversion as well as by the addition of a DAPI-positive segment on its long arm telomere distal to the PAR (Figure S3; see online Text S1); these features are responsible for the submetacentric appearance of the Y chromosome (Figure 1) in this specimen. A morphologically even more conspicuous Y chromosome variant was detected in “Max(2)” which exhibited strong FISH-signals for both *DAZ* and *CDY* in pericentromeric positions, and the presence of an additional *CDY*-signal on the subtelomeric short arm (Figure 1). Compared to the size of a “normal” chimpanzee Y chromosome, that of “Max(2)” showed a considerable increase in total length (Figure S4). To further cytogenetically dissect the subchromosomal structure of this chromosome we employed FISH analysis using DNA probes specific for ampliconic and X-degenerate genes on the Y chromosome (Figure 2). Our FISH results clearly showed that the Y chromosome of “Max(2)” exhibits a drastic increase of signals for *DUXY* sequences representing segmental duplications mapping in human Yq11.1/Yq11.21 [11], [12]. In addition, a signal increase for ampliconic *TSPY* and *RBMY* genes was visible in the Y chromosome long arm of “Max(2)”. With the exception of *USP9Y* that maps close to the centromere, the X-degenerate genes maintain their expected location in the distal half of Yq proximal to the PAR. Although *USP9Y* is thought to be required for spermatogenesis in human males [13], [14] it seems that *USP9Y* is dispensable in chimpanzees, as the two chimpanzee Y chromosomes sequenced both carry inactive forms of USP9Y [7]–[9], [15]–[17]. Thus, the translocation of *USP9Y* close to the centromere on the Y chromosome of “Max(2)” may be of no relevance. The finished MSY sequence of the index specimen “Clint” [9], enabled us to schematically map the *DAZ* and *CDY* loci on “Clint's” Y chromosome (Figure 1) providing yet another variant to our chimpanzee sample.

**Figure 3:**
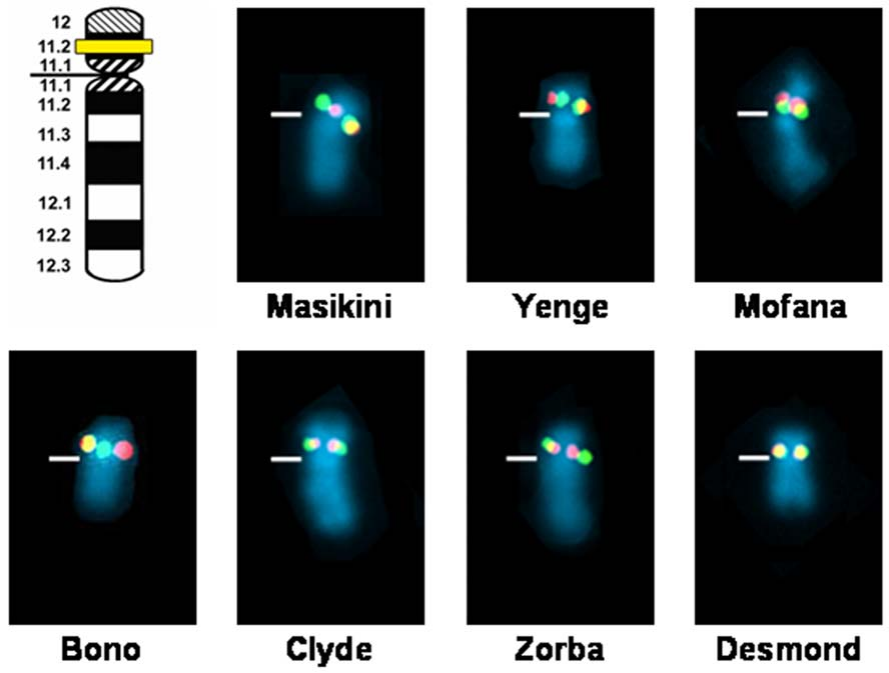
FISH mapping of ampliconic genes *DAZ* and *CDY* in seven Y chromosome lineages of bonobo. For each Y chromosome lineage the FISH signal pattern for *DAZ* (red) and *CDY* (green) is shown. Yellow signals results from overlapping of red and green signals. Centromeres are marked by horizontal lines. Band nomenclature follows the ISCN [49].

In marked contrast to the situation in *P. troglodytes*, no variation in copy number or location was detected for either *DAZ* or *CDY* on the Y chromosomes of 16 male bonobo (*P. paniscus*) specimens, representing seven wild-born bonobos (Figure S2; Table S2). In all animals investigated, *DAZ* and *CDY* map to Yp11.2 with mostly overlapping single FISH signals for both genes (Figure 3). The detection of a single signal for *DAZ* is consistent with the number of *DAZ* genes reported in 10 male bonobos [10]. Additionally, no variation was found in the X-degenerate genes (proximal to distal) *USP9Y*, *DDX3Y* (formerly *DBY*), *UTY*, *KAL*, *AMELY*, *PRKY*, and most distally *SHOX* (the PAR-gene) that are arranged on distal Yq of the bonobos. These results are in agreement with the linear stability of X-degenerate genes for both bonobo and chimpanzee [3], [5].

By comparing the Y chromosome arrangement of MSY genes between 11 chimpanzee and seven bonobo male lineages, we conclude: (i) There is high variation for ampliconic fertility genes *DAZ* and *CDY* among the chimpanzees. Of 11 chimpanzee Y-chromosomal lines, 10 variants were detected. In contrast, the fertility genes on a morphologically stable submetacentric Y chromosome were invariant among seven bonobo Y-chromosomal lines. (ii) There is minimal variation for ampliconic genes *RBMY* and *TSPY* among both chimpanzees and bonobos. That said, all bonobo Y chromosomes share an amplification of *RBMY* sequences in their proximal long arm. (iii) X-degenerate single copy genes (with exception of *USP9Y*) show stable positions on all bonobo and chimpanzee Y chromosomes investigated. Although sample size is limited, especially in our invariant bonobo sample, it should be noted that the global captive bonobo population is derived from only 35 specimens –18 males and 17 females. Of these 18 males, two potential founders have still to reproduce [18], [19]. All founders and potential founders in this population are considered to be unrelated to each other since they originated from at least four distinct populations located east to west across the bonobos' range in the Democratic Republic of the Congo (DRC)[20]. Also, wild-caught bonobos do not appear to have come from severely inbred populations with high levels of individual homozygosity [19], [20].

The natural distribution of bonobos is confined to a single area - Cuvette Centrale in the centre of the Congo Basin in the DRC [20]. This remote region of moist evergreen forests is encircled by the Congo River in the North, and the Kasai and Sankuru rivers in the South [21]. In contrast, three to four subspecies [21], [22] of chimpanzee are recognized, distributed across semideciduous forests in Central Africa [23]. Given these data one could argue that the invariant bonobo Y chromosome in our sample is the result of a founder effect, while the Y-variants in chimpanzee simply reflect subspecific variation. The latter is considered unlikely given that the number of chimpanzee Y chromosome variants detected exceeds the recognition of geographic variants and both “Hans” and “Moritz” (wild-caught in West-Africa and are attributable to *P. t. verus*), present markedly different Y-variants. In other parts of the genome there is also considerable genetic variability within chimpanzee main subpopulations [24] while, in contrast, bonobos are less polymorphic than each of the chimpanzee subpopulations [25]. Thus, loss of genetic variation by founder effect or genetics drift must further be considered as a possible explanation in the bonobo. We posit rather that the geographic isolation by the Congo River [26] probably permitted the establishment of different social systems in *P. troglodytes* and *P. paniscus* promoted, in part, by ecological and behavioural adaption [23].

Male chimpanzees remain in their natal communities, and establish dominance hierarchies with a clear alpha male. Sexually receptive females show conspicuous periovulatory swellings and mate promiscuously [27]–[30]. Hormonal patterns indicate that they ovulate when they are maximally tumescent, and males can therefore monitor female receptivity [31]. Under such conditions the opportunistic mating strategy in chimpanzee communities offers an opportunity for sperm competition in this species [32]–[34]. In contrast, the high social status of females in bonobo communities is unique to this species of *Pan* [35], [36] which, coupled to concealed ovulation, could allow greater female choice [37], and this may act as an evolutionary counterstrategy that diminishes sexual selection via sperm competition. This view is further supported by the observation that relative to chimpanzees, adult bonobos show reduced sex dimorphism in both body size [38] and the canine teeth [39], [40]. In addition, adult testosterone levels of male bonobos are much lower than those of adult chimpanzees [41], [42]. As a consequence spermatogenic gene variation might be low in modern male bonobos.

We conclude that, although chimpanzee and bonobo both show polyandrous mating behaviour with potentially high levels of sperm competition [27], [43]–[45], the contrasting patterns of Y-chromosomal variation in these closely related species might have an explanation in the context of their markedly different social structures. In chimpanzees, multiple males copulate with a receptive female during a short period of visible anogenital swelling, and this may place significant selection on fertility genes. In bonobos, however, female mate choice may make sperm competition redundant (leading to monomorphism of fertility genes), since ovulation in this species is concealed by the prolonged anogenital swelling, and because female bonobos can occupy high-ranking positions in the group and are thus able to determine mate choice more freely. We may speculate that the evolutionary history of a primate species Y chromosome is not simply encrypted in its DNA sequences but is shaped by social and behavioural circumstances. It is interesting that FISH studies in gorillas and orangutans similarly failed to detect intra-species variation in spermatogenesis genes (Greve et al., in preparation). It is apparent that monoandrous mating behaviour in gorillas [45], as well as the preference of female mate choice in orangutans [46], similarly diminishes sperm competition thus mirroring the situation in bonobo.

## Material and Methods

### Blood samples

Peripheral blood samples from all chimpanzee and bonobo specimens used in our studies were provided by zoo physicians. Details about the origin and status of the chimpanzee and bonobo specimens used in our study are presented in Tables S1 and S2. Pedigrees tracing the genealogy of the chimpanzee and bonobo specimens to the wild-born founders are shown in Figures S1 and S2.

### Chromosome preparations

Chromosome preparations of all chimpanzee and bonobo specimens were made directly from peripheral blood lymphocytes – only in the case of the chimpanzee “Sascha” from a lymphoblastoid cell line established from peripheral blood in our lab – according to standard methods with minor modifications [47]. Slides carrying interphase cells and metaphase spreads were dehydrated in a series of ice-cold ethanol (70%, 90% and 100% each for 3 min) then air dried and stored at −80°C. Before using for *in situ* hybridization, the slides were dehydrated again (70%, 90% and 100% each for 3 min) and then air dried.

### FISH analysis

All FISH-assays were performed on metaphase and prometaphase spreads following Schempp et al [2]. Prior to FISH, the slides were treated with RNase followed by pepsin digestion as described [48]. Chromosome *in situ* suppression (CISS) was applied to gene clones listed in Table S3. For two-color detection, double-hybridization experiments were performed with biotinylated and digoxigenin (DIG)-labeled probes. Biotinylated probes were detected with FITC-conjugated avidin and DIG-labeled probes with anti-DIG-mouse antibodies (Sigma) followed by TRITC-conjugated goat anti-mouse antibodies (Sigma). After FISH the slides were counterstained with DAPI (4′,6-doamidino-2-phenolindole; 0.14 μg/ml) and mounted in Vectashield (Vector Laboratories). Preparations were evaluated using a Zeiss Axiophot epifluorescence microscope equipped with single-bandpass filters for excitation of red, green, and blue (Chroma Technologies, Brattleboro, VT). During exposures, only excitation filters were changed allowing for pixel-shift-free image recording. Images of high magnification and resolution were obtained using a black-and-white CCD camera (Photometrics Kodak KAF 1400; Kodak, Tucson, AZ) connected to the Axiophot. Camera control and digital image acquisition involved the use of an Apple Macintosh Quadra 950 computer.

## Supporting Information

Figure S1 Father-son pedigrees allowing us to trace back the chimpanzees investigated to eleven wild-born males. With the exception of “Max(2)”, paternity is assured.
(0.09 MB TIF)

Figure S2 Father-son pedigrees allowing us to trace back the bonobos investigated to seven wild-born males. Paternity is assured for all male bonobos.
(0.08 MB TIF)

Figure S3 Illustration of the structural Y chromosome variation of the chimpanzee “Moritz”. The picture on the left shows a single signal for *DAZ* (green) in the short arm of the submetacentric Y chromosome. The signals for the pseudoautosomal gene *SHOX* (red) map in subtelomeric positions on chromosomes Y and X. The picture on the right shows that the location of ampliconic *RBMY* (green) and *TSPY* (red; appearing yellow because of the signal overlapping with the green *RBMY* signals) is exclusively in the proximal long arm of the Y chromosome of “Moritz” (see Text S1). Centromeres are marked by white bars.
(0.48 MB TIF)

Figure S4 Illustration of the increase in length of the metacentric Y chromosome of the chimpanzee ”Max(2)” compared to the Y chromosome of “Bimbo”. While the X chromosomes of “Max(2)” and “Bimbo” are of comparable size, the Y chromosome of “Max(2)” shows a considerable increase of length, notably in the short arm when compared to the Y chromosome of “Bimbo”. FISH with *SHOX* (red) was applied as a marker for the pseudoautosomal region assigned to the telomeres of the Y chromosome long arm and the X chromosome short arm in the chimpanzee. Centromeres are marked by white bars.
(0.04 MB DOC)

Table S1 Common chimpanzee (*Pan troglodytes*) individuals.
(0.04 MB DOC)

Table S2 Bonobo (*Pan paniscus*) individuals.
(0.04 MB DOC)

Table S3 Gene clones used for FISH.
(0.04 MB DOC)

Text S1 Structural Y chromosome alterations in wild-born chimpanzee.
(0.02 MB DOC)

## References

[1] Weber B, Schempp W, Wiesner H (1986). An evolutionary conserved early replicating segment on the sex chromosomes of man and the great apes.. Cytogenet Cell Genet.

[2] Schempp W, Binkele A, Arnemann J, Gläser B, Ma K (1995). Comparative mapping of YRRM- and TSPY-related cosmids in man and hominoid apes.. Chromosome Res.

[3] Gläser B, Grützner F, Willmann U, Stanyon R, Arnold N (1998). Simian Y chromosomes: species-specific rearrangement of DAZ, RBM, and TSPY versus contiguity of PAR and SRY.. Mammalian Genome.

[4] Lahn BT, Page DC (1999). Four evolutionary strata on the human X chromosome.. Science.

[5] Gläser B, Grützner F, Taylor K, Schiebel K, Meroni G (1997). Comparative mapping of Xp22 genes in hominoids – evolutionary linear stability of their Y homologues.. Chromosome.

[6] Skaletsky H, Kuroda-Kawaguchi T, Minx PJ, Cordum HS, Hillier I (2003). The male-specific region of the human Y chromosome is a mosaic of discrete sequence classes.. Nature.

[7] Hughes JF, Skaletsky H, Pyntikova T, Minx PJ, Graves T (2005). Conservation of Y-linked genes during human evolution revealed by comparative sequencing in chimpanzee.. Nature.

[8] Kuroki Y, Toyoda A, Noguchi H, Taylor TD, Itoh T (2006). Comparative analysis of chimpanzee and human Y chromosomes unveils complex evolutionary pathway.. Nature Genet.

[9] Hughes JF, Skaletsky H, Pyntikova T, Graves TA, van Daalen SKM (2010). Chimpanzee and human Y chromosomes are remarkably divergent in structure and gene content.. Nature.

[10] Yu YH, Lin YW, Yu JF, Schempp W, Yen PH (2008). Evolution of DAZ gene and the AZFc region on primate Y chromosomes.. BMC Evolutionary Biology.

[11] Kirsch S, Münch C, Jiang Z, Cheng Z, Chen L (2008). Evolutionary dynamics of segmental duplications from human Y-chromosomal euchromatin/heterochromatin transition regions.. Genome Res.

[12] Schmidt J, Kirsch S, Rappold GA, Schempp W (2009). Complex evolution of a Y-chromosomal double homeobox 4 (DUX4)-related gene family in hominoids.. PLOS ONE 4(4), e5288 (2009).

[13] Brown GM, Furlong RA, Sargent CA, Erickson RP, Longepied G (1998). Characterisation of the coding sequence and fine mapping of the human DFFRY gene and comparative expression analysis and mapping to the Sxrb interval of the mouse Y chromosome of the Dffry gene.. Hum Mol Genet.

[14] Sun C, Skaletsky H, Birren B, Devon K, Tang Z (1999). An azoospermic man with a *de novo* point mutation in the Y-chromosomal gene USP9Y.. Nature Genet.

[15] Tyler-Smith C, Howe K, Santos FR (2006). The rise and fall of the ape Y chromosome?. Nature Genet.

[16] Perry GH, Tito RY, Verrelli BC (2007). The evolutionary history of human and chimpanzee Y-chromosome gene loss.. Mol Biol Evol.

[17] Goto H, Peng L, Makova KD (2009). Evolution of X-degenerate Y chromosome genes in greater apes: Conservation of gene content in human and gorilla, but not chimpanzee.. J Mol Evol.

[18] Van Coillie S, Galbusera P, Roeder AD, Schempp W, Stevens JMG (2008). Molecular paternity determination in captive bonobos and the impact of inbreeding on infant mortality.. Animal Conservation.

[19] Pereboom JJM, StevensJMG (2008). International Studbook for bonobo *Pan paniscus* Schwarz 1929..

[20] Reinartz GE, Karron JD, Phillips RB, Weber JL (2000). Patterns of microsatellite polymorphism in the range-restricted bonobo (*Pan paniscus*): considerations for interspecific comparison with chimpanzees (*P. troglodytes*).. Mol Ecol.

[21] Eriksson J, Hohmann G, Boesch C, Vigilant L (2004). Rivers influence the population genetic structure of bonobos (*Pan paniscus*).. Mol Ecol.

[22] Gonder MK, Oates JF, Disotell TR, Forstner MR, Morales JC (1997). A new West African chimpanzee subspecies?. Nature.

[23] Furuichi T (2009). Factors underlying party size differences between chimpanzees and bonobos: a review and hypotheses for future study.. Primates.

[24] Morin PA, Moore JJ, Chakraborty R, Jin L, Goodall J (1994). Kin selection, social structure, gene flow, and the evolution of chimpanzees.. Science.

[25] Yu N, Jensen-Seaman MI, Chemnick L, Kidd JR, Deinard AS (2003). Low nucleotide diversity in chimpanzees and bonobos.. Genetics.

[26] Myers Thompson JA (2003). A model of the biogeographical journey from proto-pan to *Pan paniscus*.. Primates.

[27] Goodall J (1986). The Chimpanzees of Gombe: Patterns of Behavior..

[28] Nishida T, Hamburg DA, McCown ER (1979). The Social Structure of Chimpanzees of the Mahale Mountains.. The Great Apes.

[29] Tutin CEG (1980). Reproductive behaviour of wild chimpanzees in the Gombe National Park, Tanzania.. Journal of Reproduction and Fertility.

[30] Boesch C (1996). Social Grouping in Tai Chimpanzees..

[31] Deschner T, Heistermann M, Hodges K, Boesch C (2003). Timing and probability of ovulation in relation to sex skin swelling in wild West African chimpanzees, *Pan troglodytes verus*.. Animal Behaviour.

[32] Harcourt AH, Harvey PH, Larson SG, Short RV (1981). Testis weight, body weight, and breeding system in primates.. Nature.

[33] Hasegawa T, Hiraiwa-Hasegawa M (1990). Sperm Competition and Mating Behavior..

[34] Constable JL, Ashley M, Goodall J, Pusey AE (2001). Noninvasive paternity assignment in Gombe chimpanzees.. Mol Ecol.

[35] Kano T (1992). The last ape: Pygmy chimpanzee Behaviour and Ecology..

[36] Parish AR (1994). Sex and food control in the uncommon chimpanzee: how bonobo females overcome a phylogenetic legacy of male dominance.. Ethol Sociobiol.

[37] Marvan R, Stevens JMG, Roeder AD, Mazura I, Bruford MW (2005). Male dominance rank, mating and reproductive success in captive bonobos (*Pan paniscus*).. Folia Primatol.

[38] Zihlman AL, Cramer DL (1978). Skeletal differences pygmy (*Pan paniscus*) and common chimpanzee (*Pan troglodytes*).. Folia Primatol.

[39] Zihlman AL, Cronin JE, Cramer DL, Sarich VM (1978). Pygmy chimpanzee as a possible prototype for the common ancestor of humans, chimpanzees and gorilla.. Nature.

[40] Begun DR, Deane AS (2005). Reduced canine sexual dimorphism in *Pan paniscus*: A morphometric approach to canine sexing in hominoids using high resolution polynomial curve fitting (HR-PCF).. Am J Phys Anthropol.

[41] Sannen A, Heisterman M, van Elsacker L, Mohle U, Eens M (2003). Urinary testosterone metabolite levels on bonobos: a comparison with chimpanzees in relation to social system.. Behaviour.

[42] Dixson AF, Anderson MJ (2004). Sexual behaviour, reproductive physiology and sperm competition in male mammals.. Physiology and Behavior.

[43] Nishida T (1968). The social group of wild chimpanzees in the Mahale Mountains.. Primates.

[44] Kano T (1982). The social group of pygmy chimpanzees (*Pan paniscus*) of Wamba.. Primates.

[45] Dixson AF (1998). Primate Sexuality: Comparative Studies of the Prosimians, Monkeys, Apes and Human Beings..

[46] Delgado RA, van Schaik CP (2000). The behavioral ecology and conservation of the orangutan (*Pongo pygmaeus*): a tale of two islands.. Evol Anthropology.

[47] Schempp W, Meer B (1983). Cytologic evidence for three human X-chromosomal segments escaping inactivation.. Hum Genet.

[48] Ried T, Baldini A, Rand TC, Ward DC (1992). Simultaneous visualization of seven different DNA probes by *in situ* hybridization using combinatorial fluorescence and digital imaging microscopy.. Proc Natl Acad Sci USA.

[49] ISCN (2009). An International System for Human Cytogenetic Nomenclature..

