# Correction: Y Chromosomal Variation Tracks the Evolution of Mating Systems in Chimpanzee and Bonobo

**DOI:** 10.1371/annotation/14d47e6a-400a-429e-a487-6dd375e04632

**Published:** 2010-11-15

**Authors:** Felix Schaller, Antonio M. Fernandes, Christine Hodler, Claudia Münch, Juan J. Pasantes, Wolfram Rietschel, Werner Schempp

Table S1 was incorrectly published as Figure S4. Please view the correct Figure S4 here: 
