# Supplementary figures and images for: Y Chromosomal Variation Tracks the Evolution of Mating Systems in Chimpanzee and Bonobo
Source: PLoS One. 2010 Sep 1;5(9):e12482. doi: 10.1371/journal.pone.0012482 (PMC2931694; doi:10.1371/journal.pone.0012482)

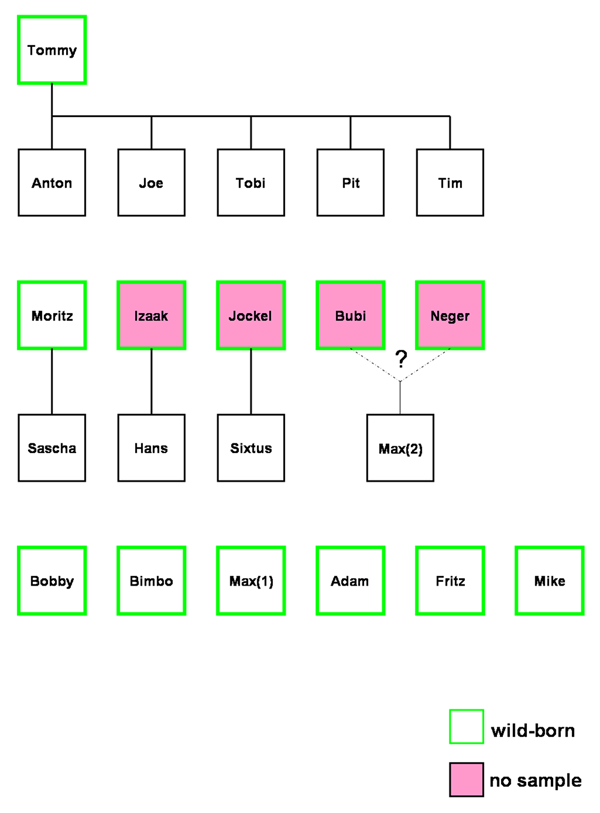

Supplement: Figure S1 — Father-son pedigrees allowing us to trace back the chimpanzees investigated to eleven wild-born males. With the exception of “Max(2)”, paternity is assured. (0.09 MB TIF) [file pone.0012482.s001.tif]

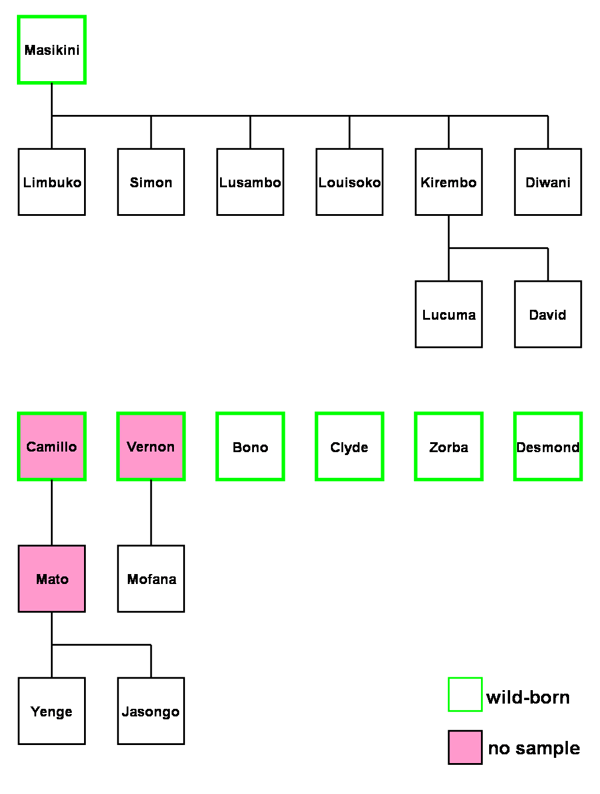

Supplement: Figure S2 — Father-son pedigrees allowing us to trace back the bonobos investigated to seven wild-born males. Paternity is assured for all male bonobos. (0.08 MB TIF) [file pone.0012482.s002.tif]

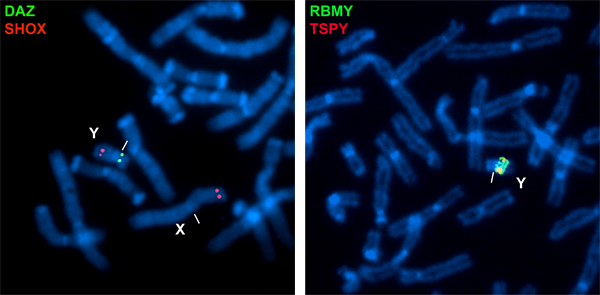

Supplement: Figure S3 — Illustration of the structural Y chromosome variation of the chimpanzee “Moritz”. The picture on the left shows a single signal for DAZ (green) in the short arm of the submetacentric Y chromosome. The signals for the pseudoautosomal gene SHOX (red) map in subtelomeric positions on chromosomes Y and X. The picture on the right shows that the location of ampliconic RBMY (green) and TSPY (red; appearing yellow because of the signal overlapping with the green RBMY signals) is exclusively in the proximal long arm of the Y chromosome of “Moritz” (see Text S1). Centromeres are marked by white bars. (0.48 MB TIF) [file pone.0012482.s003.tif]
