# Supplementary material for: Y Chromosomal Variation Tracks the Evolution of Mating Systems in Chimpanzee and Bonobo
Source: PLoS One. 2010 Sep 1;5(9):e12482. doi: 10.1371/journal.pone.0012482 (PMC2931694; doi:10.1371/journal.pone.0012482)
Supplement: Figure S4 — Illustration of the increase in length of the metacentric Y chromosome of the chimpanzee ”Max(2)” compared to the Y chromosome of “Bimbo”. While the X chromosomes of “Max(2)” and “Bimbo” are of comparable size, the Y chromosome of “Max(2)” shows a considerable increase of length, notably in the short arm when compared to the Y chromosome of “Bimbo”. FISH with SHOX (red) was applied as a marker for the pseudoautosomal region assigned to the telomeres of the Y chromosome long arm and the X chromosome short arm in the chimpanzee. Centromeres are marked by white bars. (0.04 MB DOC) [file pone.0012482.s004.doc]

**Table S1: Common chimpanzee (*Pan troglodytes)* individuals**

| **Name** | **Studbook #** | **Zoo** | **Birth Date** | **Notes** |
| --- | --- | --- | --- | --- |
| Adam | 11727 | Leintalzoo, Schwaigern | wb ~1978 |  |
| Anton |  | Schwabenpark, Welzheim | ? | Son of Tommy |
| Bimbo |  | Schwabenpark, Welzheim | wb ? |  |
| Bobby |  | Schwabenpark, Welzheim | wb ? |  |
| Fritz |  | Schwabenpark, Welzheim | wb ? |  |
| Hans * | 11508 | TNO, Netherlands | 07.09.1974 | Son of Isaak wb |
| Joe |  | Schwabenpark, Welzheim | ? | Son of Tommy |
| Max(1) |  | Schwabenpark, Welzheim | wb ? |  |
| Max(2) | 12914 | Leintalzoo, Schwaigern | 10.01.1997 | Son of Bubi wb ? |
| Mike |  | Schwabenpark, Welzheim | wb ? |  |
| Moritz * | 10920 | Wilhelma, Stuttgart | wb ~1932 | Nigeria |
| Pit |  | Schwabenpark, Welzheim | ? | Son of Tommy |
| Sascha * | 12527 | Schwabenpark, Welzheim | 20.12.1990 | Son of Moritz |
| Sixtus | 12971 | Schwabenpark, Welzheim | 29.11.1997 | Son of Jockel wb |
| Tim |  | Schwabenpark, Welzheim | ? | Son of Tommy |
| Tobi |  | Schwabenpark, Welzheim | ? | Son of Tommy |
| Tommy |  | Schwabenpark, Welzheim | wb ? |  |

European Studbook for the Chimpanzee *Pan troglodytes* (Frands Carlsen, Copenhagen Zoo, Frederiksberg, Denmark, 2007).

***** *Pan troglodytes verus*

wb: wild-born
