# Supplementary material for: Y Chromosomal Variation Tracks the Evolution of Mating Systems in Chimpanzee and Bonobo
Source: PLoS One. 2010 Sep 1;5(9):e12482. doi: 10.1371/journal.pone.0012482 (PMC2931694; doi:10.1371/journal.pone.0012482)
Supplement: Table S1 — Common chimpanzee (Pan troglodytes) individuals. (0.04 MB DOC) [file pone.0012482.s005.doc]

**Table S1: Common chimpanzee (*Pan troglodytes)* individuals**

| **Name** | **Studbook #** | **Zoo** |
| --- | --- | --- |
| Adam | 11727 | Leintalzoo, Schwaigern |
| Anton |  | Schwabenpark, Welzheim |
| Bimbo |  | Schwabenpark, Welzheim |
| Bobby |  | Schwabenpark, Welzheim |
| Fritz |  | Schwabenpark, Welzheim |
| Hans * | 11508 | TNO, Netherlands |
| Joe |  | Schwabenpark, Welzheim |
| Max(1) |  | Schwabenpark, Welzheim |
| Max(2) | 12914 | Leintalzoo, Schwaigern |
| Mike |  | Schwabenpark, Welzheim |
| Moritz * | 10920 | Wilhelma, Stuttgart |
| Pit |  | Schwabenpark, Welzheim |
| Sascha * | 12527 | Schwabenpark, Welzheim |
| Sixtus | 12971 | Schwabenpark, Welzheim |
| Tim |  | Schwabenpark, Welzheim |
| Tobi |  | Schwabenpark, Welzheim |
| Tommy |  | Schwabenpark, Welzheim |

***** *Pan troglodytes verus*

European Studbook for the Chimpanzee *Pan troglodytes* (Frands Carlsen, Copenhagen Zoo, Frederiksberg, Denmark, 2007).
