# Supplementary material for: Y Chromosomal Variation Tracks the Evolution of Mating Systems in Chimpanzee and Bonobo
Source: PLoS One. 2010 Sep 1;5(9):e12482. doi: 10.1371/journal.pone.0012482 (PMC2931694; doi:10.1371/journal.pone.0012482)
Supplement: Table S2 — Bonobo (Pan paniscus) individuals. (0.04 MB DOC) [file pone.0012482.s006.doc]

**Table S2: Bonobo (*Pan paniscus*) individuals**

| **Name** | **Studbook #** | **Zoo** | **Birth Date** | **Notes** |
| --- | --- | --- | --- | --- |
| Bono | 102 | Zoo Frankfurt | wb ~1977 | founder |
| Clyde | 97 | Zoo Cologne | wb ? | founder |
| David | 277 | Wilhelma, Stuttgart | 27.07.2001 | Son of Masikini |
| Diwani | 215 | Wilhelma, Stuttgart | 11.08.1996 | Son of Masikini |
| Desmond | 57 | Planckendael, Belgium | wb ~1970 | founder |
| Jasongo | 159 | Twycross, UK | 02.08.1990 | Grandson of Camillo wb ~1952 (founder) |
| Kirembo | 177 | Wilhelma, Stuttgart | 10.12.1992 | Son of Masikini |
| Limbuko | 210 | Zoo Leipzig | 04.10.1995 | Son of Masikini |
| Louisoko | 244 | Wilhelma, Stuttgart | 19.04.1998 | Son of Masikini |
| Lucuma | 294 | Wilhelma, Stuttgart | 29.10.2002 | Son of Masikini |
| Lusambo | 107 | Wilhelma, Stuttgart | 21.07.1980 | Son of Masikini |
| Masikini | 54 | Wilhelma, Stuttgart | wb ~1966 | founder |
| Mofana | 84 | Memphis, Tennessee | 16.12.1978 | Son of Vernon wb ~1970 (founder) |
| Simon | 85 | Zoo Berlin | 25.11.1979 | Son of Masikini |
| Yenge | 111 | San Diego Zoo | 25.12.1982 | Grandson of Camillo (founder) |
| Zorba | 168 | Wilhelma, Stuttgart | wb ~1980 | founder |

International Studbook for bonobo *Pan paniscus* SCHWARZ 1929 (JJM Pereboom & JMG Stevens, Royal Zoological Society of Antwerp, Antwerpen, Belgium, 2008).

wb: wild-born
