# Supplementary material for: Y Chromosomal Variation Tracks the Evolution of Mating Systems in Chimpanzee and Bonobo
Source: PLoS One. 2010 Sep 1;5(9):e12482. doi: 10.1371/journal.pone.0012482 (PMC2931694; doi:10.1371/journal.pone.0012482)
Supplement: Table S3 — Gene clones used for FISH. (0.04 MB DOC) [file pone.0012482.s007.doc]

**Table S3: Gene clones used for FISH**

| Sequence class | Gene | Clone | Reference |
| --- | --- | --- | --- |
| ampliconic | *CDY* | cos CDY-2A49 | 1 |
|  | *DAZ* | cos 6B7; cos 7F11 | 2 |
|  | *DUXY* | cos 70B12; cos 118E07 | 3 |
|  | *RBMY* | cos A5F | 2 |
|  | *TSPY* | cos 2.2133 | 4 |
| X-degenerate | *AMELY* | cos C9E | 2 |
|  | *DDX3Y* (*DBY*) | PAC 99C09 | 5 |
|  | *KAL* | cos QA8; cos 21H5 | 6 |
|  | *PRKY* | PAC 152F08; PAC 283F16 | 7 |
|  | *USP9Y* | PAC 529G14 | 5 |
|  | *UTY* | PAC 91N20 | 5 |
| pseudoautosomal | *SHOX* | cos 34F05 | 8 |

1. Kühl, H., Röttger, S., Heilbronner, H., Enders, H., Schempp, W. Loss of the Y chromosomal PAR2-region in four familial cases of satellited Y chromosomes (Yqs). Chromosome Res. 9, 215-222 (2001).
2. Taylor, K. et al. Mapping the human Y chromosome by fingerprinting cosmid clones. Genome Res. 6, 235-248 (1996).
3. Schmidt, J., Kirsch, S., Rappold, G. A., Schempp, W. Complex evolution of a Y-chromosomal double homeobox 4 (DUX4)-related gene family in hominoids. PLoS ONE 4(4): e5288. doi:10.1371/journal.pone.0005288 (2009).
4. Taylor, K., Ulinowski, Z., Wolfe, J. Contig assembly of cosmid clones from the Y chromosome. Cytogenet. Cell. Genet. 58, 2096 (1991).
5. Wimmer, R., Kirsch, S., Weber, A., Rappold, G. A., Schempp, W. The azoospermia region AZFa: an evolutionary view. Catogenet. Genome Res. 99, 146-150 (2002).
6. Gläser, B. et al. Comparative mapping of Xp22 genes in hominoids – evolutionary linear instability of their Y homologues. Chromosome Res. 5, 167-176 (1997).
7. Schiebel, K. et al. FISH localization of the human Y-homolog of protein kinase PRKX (PRKY) to Yp11.2 and two pseudogenes to 15q26 and Xq12 – q13. Cytogenet. Cell Genet. 76, 49-52 (1997).
8. Rao, E. et al. Pseudoautosomal deletions encompassing a novel homeobox gene cause growth failure in idiopathic short stature and Turner syndrome. Nature Genet. 16, 54-63 (1997).
