# Supplementary material for: Y Chromosomal Variation Tracks the Evolution of Mating Systems in Chimpanzee and Bonobo
Source: PLoS One. 2010 Sep 1;5(9):e12482. doi: 10.1371/journal.pone.0012482 (PMC2931694; doi:10.1371/journal.pone.0012482)
Supplement: Text S1 — Structural Y chromosome alterations in wild-born chimpanzee. (0.02 MB DOC) [file pone.0012482.s008.doc]

**Text S1**

*Structural Y chromosome alterations in wild-born chimpanzee „Moritz“*

The same addition of a DAPI-positive segment distal to the PAR was detected on the Y chromosome as well as the X chromosome of “Moritz” (Figure S3). This additional DAPI-positive segment may represent a heterochromatic cap that is part of the PAR specifically in chimpanzee “Moritz”. Interestingly, the analogous extension of the X-Y pairing region is constitutively present on the gorilla X and Y chromosomes [1,2]. Furthermore, and in contrast to the Y-pericentromeric locations of ampliconic *TSPY* and *RBMY* genes in chimpanzees “Bobby”, “Bimbo”, and “Fritz” [3,4], both genes map exclusively to the proximal Y chromosome long arm of “Moritz” (Figure S3).

1. Weber B, Schempp W, Wiesner H (1986) An evolutionary conserved early replicating segment on the sex chromosomes of man and the great apes. Cytogenet Cell Genet 43: 72-78.
2. Gläser B, Grützner F, Taylor K, Schiebel K, Meroni G, et al. (1997) Comparative mapping of Xp22 genes in hominoids – evolutionary linear stability of their Y homologues. Chromosome Res 5: 167- 176.
3. Schempp W, Binkele A, Arnemann J, Gläser B, Ma K, et al. (1995) Comparative mapping of YRRM- and TSPY-related cosmids in man and hominoid apes. Chromosome Res 3: 227-234.
4. Gläser B, Grützner F, Willmann U, Stanyon R, Arnold N, et al. (1998) Simian Y chromosomes: species-specific rearrangement of DAZ, RBM, and TSPY versus contiguity of PAR and SRY. Mammalian Genome 9: 226-231.
