# Supplementary figures and images for: Correction: Y Chromosomal Variation Tracks the Evolution of Mating Systems in Chimpanzee and Bonobo
Source: PLoS One. 2010 Nov 15;5(11):10.1371/annotation/14d47e6a-400a-429e-a487-6dd375e04632. doi: 10.1371/annotation/14d47e6a-400a-429e-a487-6dd375e04632 (PMC2982988; doi:10.1371/annotation/14d47e6a-400a-429e-a487-6dd375e04632)

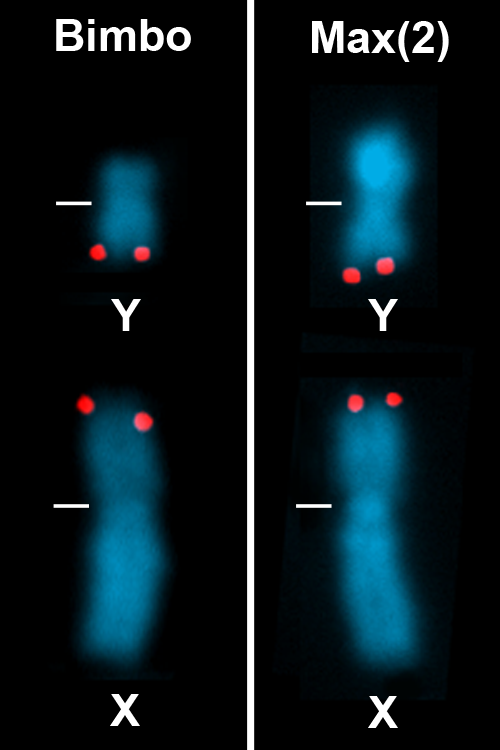

Supplement: Supplementary file 1 [file pone.14d47e6a-400a-429e-a487-6dd375e04632.s001.tif]
